# Field estimates of parentage reveal sexually antagonistic selection on body size in a population of *Anolis* lizards

**DOI:** 10.1002/ece3.2443

**Published:** 2016-09-09

**Authors:** Mary C. Duryea, Patrick Bergeron, Zachary Clare‐Salzler, Ryan Calsbeek

**Affiliations:** ^1^ Department of Biological Sciences Dartmouth College Hanover NH USA; ^2^ Present address: Department of Biology Lund University Lund Sweden; ^3^ Present address: Department of Biological Sciences Bishop's University Sherbrooke QC Canada; ^4^ Present address: Division of Biological Sciences University of Montana Missoula MT USA

**Keywords:** fecundity, natural selection, reproductive success, reptiles, sexual conflict, sexual selection

## Abstract

Sexual dimorphism evolves when selection favors different phenotypic optima between the sexes. Such sexually antagonistic selection creates intralocus sexual conflict when traits are genetically correlated between the sexes and have sex‐specific optima. Brown anoles are highly sexually dimorphic: Males are on average 30% longer than females and 150% heavier in our study population. Viability selection on body size is known to be sexually antagonistic, and directional selection favors large male size whereas stabilizing selection constrains females to remain small. We build on previous studies of viability selection by measuring sexually antagonistic selection using reproductive components of fitness over three generations in a natural population of brown anoles. We estimated the number of offspring produced by an individual that survived to sexual maturity (termed RS^V^), a measure of individual fitness that includes aspects of both individual reproductive success and offspring survival. We found directional selection on male body size, consistent with previous studies of viability selection. However, selection on female body size varied among years, and included periods of positive directional selection, quadratic stabilizing selection, and no selection. Selection acts differently in the sexes based on both survival and reproduction and sexual conflict appears to be a persistent force in this species.

## 1 Introduction

In a sexually dimorphic species, selection often favors different optima between the sexes (Lande, 1980; Roff, 1997). Phenotypic divergence in traits related to male and female fitness occurs when natural, sexual, or fecundity selection pull the sex‐specific means of a heritable phenotype away from each other. For traits that have a positive genetic correlation between the sexes, selection that moves one sex toward its phenotypic optimum displaces the other sex from its fitness optimum. The result of such sexually antagonistic selection acting on traits that have a positive genetic correlation is called intralocus sexual conflict (Bonduriansky & Chenoweth, 2009).

Body size is one of the most frequently cited targets of natural and sexual selection (Andersson, 1994; Lande, 1980), and it is often subject to sexually antagonistic selection (Cox & Calsbeek, 2009). This is because body size is important in a wide variety of contexts ranging from species sorting (Pfenning & Pfenning, 2012; Schluter, 2000), to territoriality (Parker, 1974; Maynard Smith, 1974) and to mate choice (Andersson, 1994). In many sexually dimorphic species, body size is also a trait that is associated with fitness. As such, previous studies have demonstrated a strong link between body size variation and intralocus genetic conflict across a wide range of species (Bonduriansky & Chenoweth, 2009; Shine, 1989; Slatkin, 1984). Male‐biased sexual dimorphism (i.e., males larger than females) often arises as a result of strong sexual selection, either through male–male competition or female choice (Andersson, 1994; Trivers, 1972). Larger male body size may confer an advantage if larger males are able to outcompete other males, or if they are preferred by females. However, if there are costs for larger body size in females, selection may act antagonistically on female body size, as larger females may require greater resources, take more time to develop, or they may suffer reduced viability (Bonduriansky & Chenoweth, 2009). Therefore, sexually antagonistic selection could result in intralocus sexual conflict on body size.

Brown anoles exhibit a pronounced sexual dimorphism in body size: Males are an average of 30% longer and 150% heavier than females (Cox & Calsbeek, 2010c; Stamps, 1999). Previous studies have documented sexually antagonistic selection on body size using individual viability as a measure of fitness. Cox and Calsbeek (2010c) showed that males underwent directional selection for larger body size, whereas females experienced stabilizing selection on body size. Moreover, offspring sired by larger males have higher survival (Cox & Calsbeek, 2010a) and females cryptically bias offspring sex as a function of sire body size, producing more sons from larger sires (Calsbeek & Bonneaud, 2008; Cox & Calsbeek, 2010a). Here, we build on previous studies of natural selection on the brown anole, *Anolis sagrei*. We incorporate genetic parentage analysis to investigate how body size affects individual fitness through reproductive success. Specifically, we measure the production of offspring that survive to maturity (termed RS^V^)—a measure of individual fitness that includes aspects of both reproductive success and the viability of their offspring (Calsbeek, Duryea, Goedert, Bergeron, & Cox, 2015).

Because anoles are highly territorial, we expected larger males to be more successful in bouts of intrasexual competition, and to have greater access to territories and higher RS^V^ (Calsbeek & Marnocha, 2006; Stamps, 1988; Tokarz, 1995). Additionally, because larger males produce offspring with higher survival, they may show higher lifetime reproductive success due to increased offspring viability or through female preference of larger males (Cox & Calsbeek, 2010a; Eberhard, 1996). Thus, we expected larger males to have higher reproductive success and offspring with greater viability. We predicted that patterns of selection on body size that are derived from field estimates of parentage should therefore parallel patterns of selection based on viability.

Fecundity selection could likewise favor larger female body size if larger females have more resources to invest in reproduction, as is often the case in species that lay variable numbers of eggs (Cody, 1966; Olsson, Shine, Wapstra, Uivari, & Madsen, 2002). This hypothesis predicts that in contrast to males, females might experience alternative forms of viability and fecundity selection acting on body size [i.e., stabilizing viability selection (Cox & Calsbeek, 2010c) and directional fecundity selection]. However, because anoles lay individual eggs over the course of the breeding season rather than in a single clutch (Calsbeek & Bonneaud, 2008; Cox et al., 2010; Tokarz, 1995), large female body size may not confer a fecundity advantage. Laboratory studies have shown that larger females tend to lay more eggs (Warner & Lovern, 2014), but we still do not know whether such a fecundity advantage exists in wild populations of anoles. Our goals in this study were to categorize the form and estimate the strength of selection acting on male and female body size based on offspring viability. Additionally, we investigate whether reproductive measures of fitness are in line with fitness measures based on adult survival to determine whether sexually antagonistic selection is operating through multiple components of fitness.

## 2 Methods

### 2.1 Study species

The brown anole is a small, semi‐arboreal lizard that has a broad tropical and subtropical distribution. It is a member of the “trunk‐ground” ecomorph of the adaptive radiation of *Anolis* lizards and is the most common anole in The Bahamas (Losos, Warheitt, & Schoener, 1997; Williams, 1969). During the breeding season, female anoles mate with several males and store sperm in specialized structures in their reproductive tracts (Conner & Crews, 1980). *Anolis sagrei* is highly promiscuous (Calsbeek, Bonneaud, Prabhu, Manoukis, & Smith, 2007), and rates of multiple paternity in the wild are high (e.g., more than 80% of females produce offspring with multiple sires; Calsbeek & Bonneaud, 2008). Females iteratively lay one or two eggs at approximately 11‐day intervals throughout the breeding season, and total reproductive output varies significantly among individuals (Cox et al., 2010). In experimental studies, larger females have been shown to produce more eggs and prey availability positively affects individual egg mass (Warner & Lovern, 2014). Mortality in the wild is high, and most individuals tend to survive 1 year or less, making *A. sagrei* essentially an annual species (Cox & Calsbeek, 2010c).

### 2.2 Field sampling

This study was conducted on Kidd cay, a small cay connected to the main island of Great Exuma, The Bahamas (23°30′N, 75°45′W) by a cement causeway. This population has been the subject of long‐term demographic studies since 2002 (Calsbeek & Smith, 2003). Because this site is separated from the mainland by a narrow causeway, we are able to capture most individuals in the population with high reliability (Cox & Calsbeek, 2010c). For each year of this study (2005‐2008), all adult individuals were caught by slip noose or by hand. Each adult was either toe‐clipped or injected with a unique combination of colored elastomer implants (Calsbeek & Bonneaud, 2008) for unique identification and was marked with a temporary paint dot to prevent recapture. We collected phenotypic data on all captured individuals, including snout–vent length (SVL, nearest mm), mass (g), and hindlimb and forelimb length (mm). We collected a 2 mm tissue sample from the tip of the tail for subsequent genetic analyses. The following year, we censused the population and recorded individual survival using the unique toe clips or fluorescent tags as identification. Unmarked individuals were assumed to be offspring from the previous year's cohort of parents. These individuals were assigned a unique identifier, and we collected all phenotypic metrics and a tissue sample from all unmarked individuals as described above. Individuals that were captured with a previous year's marking were excluded from the offspring pool for parentage assignment. These methods were replicated for each year of the study from 2005 to 2008.

### 2.3 Genetic analysis

DNA was extracted from each individual using a DNeasy Blood and Tissue Kit (Qiagen, Inc.), following manufacturer's protocols for tissue extraction but eluting into a volume of 30 μl AE buffer to ensure high yields of DNA. We conducted PCR on each sample using primers designed to amplify 10 microsatellite markers (Table S1). Markers AAAG‐38, AAAG‐61, AAAG‐68, AAAG‐70, AAAG‐76, AAAG‐77, AAAG‐91, and AAAG‐94 were previously designed for *A. sagrei* (Bardeleben, Palchevskiy, Calsbeek, & Wayne, 2004). Markers Acar11 and Acar23 were developed using the *Anolis carolinensis* genome by Wordley, Slate, and Stapley (2010), and we verified amplification and polymorphism in our population of *A. sagrei*. Markers were grouped into two pool sets of five markers each, and we individually tagged forward primers with a fluorescent tag (Life Technology, Inc.) that uniquely identified loci as a function of fragment size‐range in each pool (Table S1). Microsatellite markers were amplified in multiplex PCR of each pool using Type‐It Kits (Qiagen, Inc.). We conducted each multiplex PCR in a 10 μl volume using 1 μl DNA template, 5 μl Master Mix (Qiagen, Inc.), 1 μl primer mix (See Table S1 for primer concentrations), and 3 μl molecular grade water. Primer concentrations were optimized to marker‐specific amplification rates based on preliminary genotyping runs. PCR products were diluted for genotyping in 18.85 μl Hi‐Di Formamide (Life Technology, Inc.) and 0.15 μl LIZ sizing standard (Life Technology, Inc.). Diluted products were genotyped on an ABI3730 Genetic Analyzer (Life Technology, Inc.), and fragment sizes were binned and verified by eye using GeneMapper software (Life Technology, Inc.).

### 2.4 Parentage analysis

We assigned maternity and paternity to each individual in each year using the previous year's cohort as candidate parents. In the few cases in which individuals survived for more than 1 year, they were also included as potential parents for the young of the year. Parentage was assigned with the program COLONY using the pairwise approach (Jone & Wang, 2010). COLONY's pairwise approach for assigning maternity and paternity uses a LOD score comparison to assign confidence (e.g., 80%–95%) to parentage calls. For each run of COLONY, we set the probability that all potential mothers or fathers were included in the dataset as 0.90 and used a “Medium” run length. We selected the sire and dam with the highest likelihood as each offspring's parents at a minimum confidence level of 80%.

### 2.5 Statistical analyses

We tested for differences in SVL between the sexes and among years using ANOVA. We tested for variation in RS^V^ (calculated as the number of offspring that survived to sexual maturity) between the sexes and among years using a generalized linear model with a log link function to account for the Poisson distribution of offspring counts. Each model included year, sex, and their interaction as effects.

Using the COLONY output, we assigned offspring to parents. Although we were not able to assign parentage to all individuals in the population, we have no reason to expect a bias in the genetic assignments because individuals were sampled exhaustively without consideration of body size, and because unassigned parentage occurred at random. Thus, our data represent an unbiased sample of this population. We used this measure of individual fitness (RS^V^: total offspring surviving to maturity, Calsbeek et al., 2015) to investigate selection on male and female body size. We also used the genetic assignment to calculate the number of mates with which each individual produced offspring as a measure of multiple mating.

We estimated relative fitness for each individual (separately by sex and year), by dividing individual fitness (RS^V^) by the population mean fitness. We standardized phenotypic traits for each year and sex to have a mean of zero and unit variance (Lande & Arnold, 1983). We estimated selection gradients from a multiple regression of standardized traits (e.g., SVL) on relative fitness (Lande & Arnold, 1983). Directional selection gradients were estimated from a model that included only linear terms. Quadratic forms of selection were estimated by doubling the value of the quadratic regression coefficients (and their associated standard errors) from models that included both linear and quadratic terms (Stinchcombe, Agrawal, Hohenlohe, Arnold, & Blows, 2008). We tested for significance of selection using a generalized linear model with a log link function to account for the Poisson distribution of RS^V^ (offspring counts). Selection was analyzed separately for each sex, due to both the high degree of sexual dimorphism in this species and our a priori hypotheses regarding differences in selection acting on male and female body size. For comparison with a previous study of viability selection in this population (Cox & Calsbeek, 2010c), we also analyzed selection on male body size separately including subadult males and excluding subadult males. Males in the range of SVL from 40 to 50 mm are generally considered subadult, although some have been shown to achieve reproductive success (Cox & Calsbeek, 2010c). Males less than 40 mm in SVL and females less than 35 mm in SVL were excluded from the dataset because these are the minimum body sizes associated with sexual maturity (Cox & Calsbeek, 2010c). To assess overall patterns of selection on body size, we pooled data from all 3 years and both sexes, and included year and sex as factors. We tested for two‐way interaction terms to assess differences in selection acting between the sexes and among years. Because most individuals in our study live for only one reproductive season, each year represents a unique selection event and these analyses were conducted only to observe the overall patterns in selection across years.

We also tested for evidence of selection on hindlimb and forelimb length for both males and females. To assess overall patterns of selection on limb length, we pooled data for all years for each sex. We standardized limb length for each sex to have a mean of zero and unit variance (Lande & Arnold, 1983). We estimated selection gradients from a multiple regression of standardized limb length on relative fitness (RS^V^).

## 3 Results

During 2005, 2006, and 2007, respectively, we collected phenotypic and genetic data for 99, 148, and 102 males and 111, 151, and 118 females. These individuals were used as potential parents in the COLONY analysis for the following year's cohort. For 2005, paternity was assigned to 146 of 234 offspring (62% of individuals) and maternity was assigned to 144 (62% of individuals). For 2006, paternity was assigned to 61 of 146 offspring (42% of individuals) and maternity was assigned to 58 of 146 offspring (40% of individuals). For 2007, paternity was assigned to 88 of 234 offspring (38% of individuals) and maternity was assigned to 76 of 234 offspring (32% of individuals). Zero paternity was observed for individuals across the range of body sizes that we sampled (Fig. S1). Thus, we see no relationship between individual body size and our ability to assign paternity.

Body size (SVL) differed significantly between the sexes (*F*_2,828_ = 1682.68; *p *<* *.0001) and among years (*F*_2,828_ = 3.95; *p *=* *.02). Individuals tended to be smaller in 2005 for both sexes (mean ± *SD* = female 42.92 ± 2.59 mm; males 54.87 ± 5.11 mm) than in 2006 (females 43.13 ± 2.45 mm; males 56.05 ± 6.06 mm) and 2007 (females 43.22 ± 2.76 mm; males 56.77 ± 6.58 mm; Table 1). The total number of assigned offspring varied significantly by sex (χ^2^ = 9.3; *p *=* *.002) and year (χ^2^ = 118.29; *p *<* *.0001; Table 1). Overall, males tended to have more assigned offspring than females for all years (Table 1). The mean number of assigned offspring for both sexes was much higher in 2005, although low estimates for 2006 and 2007 may have been partially due to our reduced ability to assign parentage for those years (Table 1).

**Table 1:** Phenotypic traits (snout–vent length [SVL] and body condition, calculated as the residuals from a regression of log_10_ body mass against log_10_ SVL) and RS^V^ (number of offspring surviving to maturity) by sex for each year of the study

	Male (mean ± *SD*; range)	Female (mean ± *SD*; range)
2005		
SVL (mm)	54.87 ± 5.11; 41–66	42.92 ± 2.59; 35–49
RS^V^	1.24 ± 1.28; 0–5	1.07 ± 1.25; 0–8
2006		
SVL (mm)	56.05 ± 6.06; 36–66	43.13 ± 2.45; 36–50
RS^V^	0.42 ± 0.71; 0–4	0.35 ± 0.68, 0–3
2007		
SVL (mm)	56.77 ± 6.58; 30–67	43.22 ± 2.76; 34–49
RS^V^	0.72 ± 1.03; 0–5	0.44 ± 0.72; 0–4

**Figure 1:**
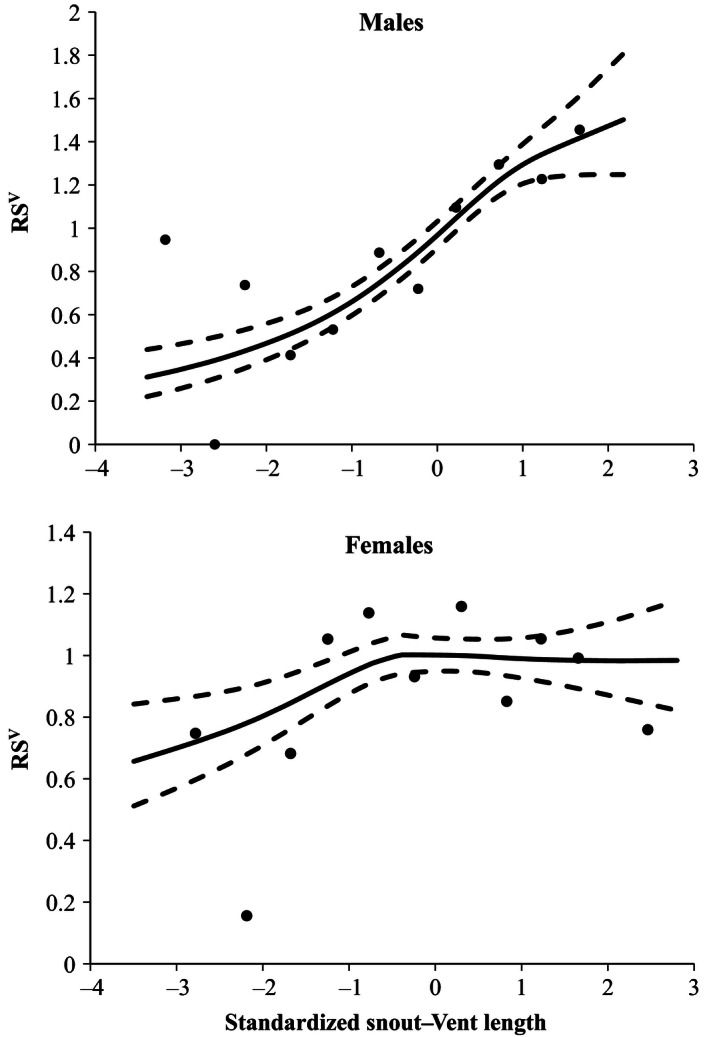
Fitness surfaces illustrating selection on male and female body size in brown anoles (*Anolis sagrei*) based on 3 years of parentage data. Solid lines show the best‐fit cubic spline, and dashed lines indicate 95% confidence intervals based on 500 bootstrap replicates. Points indicate average RS^V^ by size class intervals of 0.5 units of standardized snout–vent length

**Figure 2:**
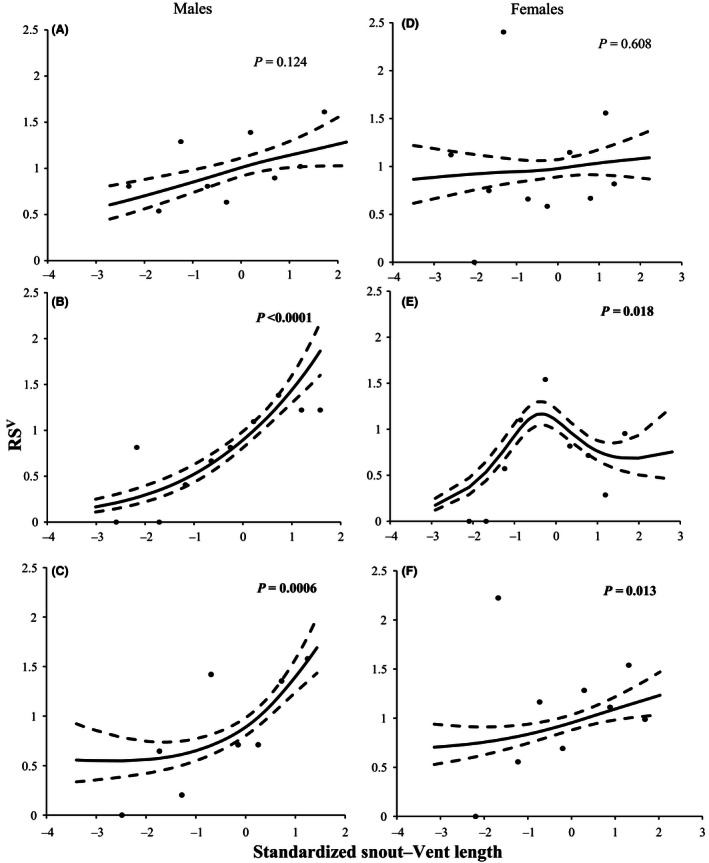
Fitness surfaces illustrating selection on male and female body size in brown anoles (*Anolis sagrei*). Solid lines show the best‐fit cubic spline, and dashed lines indicate 95% confidence intervals based on 500 bootstrap replicates. Points indicate average RS^V^ by size class intervals of 0.5 units of standardized snout–vent length. Each plot based on 1 year of parentage data, from top to bottom: 2005, 2006, and 2007. Males experienced significant directional selection in 2006 (B) and 2007 (C). Females experienced significant directional selection in 2007 (F) and stabilizing selection in 2006 (E). No evidence of selection was detected for males (A) or females (D) in 2005

When data were pooled for all years, overall patterns revealed that males were subject to directional selection on SVL (β = .28 ± .08; χ^2^ = 28.06; *p *<* *.0001, Fig. 1) and females to quadratic stabilizing selection (γ_1,1_ = −0.16 ± 0.11; χ^2^ = 6.31; *p *=* *.012, Fig. 1). Although there was not a significant interaction between year and SVL in either males (year × SVL; χ^2^ = 4.93; *p *=* *.09) or females (year × SVL; χ^2^ = 4.40; *p *=* *.11), the form of selection varied among years, particularly in females (Table 2, Fig. 2). In 2006, females experienced significant quadratic selection (Table 2, Fig. 2). In 2007, females experienced significant directional selection and marginally significant quadratic selection (Table 2, Fig. 2). By contrast, although selection on males was not significant in every year, it tended to be consistently directional when acting on SVL (Table 2, Fig. 2). The power to detect significant forms of selection may have been limited by our ability to assign paternity for some years and may indicate why no significant form of selection was found for males or females in 2005.

**Table 2:** Linear (β) and quadratic (γ) selection gradients for selection on standardized snout–vent length (SVL) based on relative RS^V^ (number of offspring that survived to maturity) and viability (V)

Year	Sex (SVL, mm)	*N* (RS^V^)	Linear selection (RS^V^)β ± 1 *SE* (*p* value)[95% CI]	Quadratic selection (RS^V^)γ ± 1 *SE* (*p* value)[95% CI]	*N* (V)	Linear selection (V)β ± 1 *SE* (*p* value)	Quadratic selection(V) γ ± 1 *SE* (*p* value)
2005	Male (≥40)	112	0.144 ± 0.097 (.1238)[−0.048, 0.337]	−0.119 ± 0.146 (.292)[−0.204, 0.085]	–	–	–
	Male (≥50)	96	0.153 ± 0.143 (.2994)[−0.130, 0.436]	−0.469 ± 0.349 (.166)[−0.580, 0.112]	–	–	–
	Female (All)	124	0.048 ± 0.110 (.608)[−0.169, 0.265]	−0.047 ± 0.147 (.681)[−0.169, 0.122]	–	–	–
2006	Male (≥40)	146	**0.366 ± 0.153 (<.0001)[0.063, 0.668]**	0.022 ± 0.310 (0.405)[−0.295, 0.318]	–	–	–
	Male (≥50)	120	**0.359 ± 0.305 (.0418)[**−**0.245, 0.962]**	−0.555 ± 0.943 (.209)[−1.211, 0.657]	–	–	–
	Female (All)	166	−0.045 ± 0.151 (.564)[−.0344, 0.254]	−**0.240 ± 0.218 (.018)[**−**0.335, 0.095]**	–	–	–
2007	Male (≥40)	119	**0.353 ± 0.154 (.0006)[0.047, 0.659]**	0.292 ± 0.348 (.519)[−0.198, 0.490]	119	**0.328 ± 0.155**	−0.148 ± 0.192
	Male (≥50)	100	**0.492 ± 0.299 (.0161)[**−**0.101, 1.085]**	0.325 ± 1.00 (.885)[−0.832, 1.157]	100	0.096 ± 0.116	−0.016 ± 0.200
	Female (All)	167	**0.198 ± 0.129 (.0131)[**−**0.057, 0.453]**	−0.160 ± 0.216 (.096)[−0.293, 0.13	161	0.085 ± 0.103	−**0.586 ± 0.182**
2008	Male (≥40)	–	–		146	−0.033 ± 0.147	0.104 ± 0.262
	Male (≥50)	–	–	–	123	0.007 ± 0.162	0.200 ± 0.254
	Female (All)	–	–	–	222	−0.084 ± 0.099	−0.186 ± 0.146
All	Male (≥40)	377	**0.286 ± 0.080 (<.0001)[0.128, 0.444]**	−0.011 ± 0.146 (0.279)[−0.150, 0.138]	264	**0.183 ± 0.049**	−0.006 ± 0.170
	Male (≥50)	316	**0.306 ± 0.144 (.0021**)**[0.023, 0.589]**	−0.407 ± 0.390 (0.067)[−0.587, 0.180]	223	0.056 ± 0.097	0.082 ± 0.158
	Female (All)	457	0.065 ± 0.079 (.1774)[−0.090, 0.219]	−**0.157 ± 0.114 (.012)[**−**0.190, 0.033]**	383	0.011 ± 0.058	−**0.241 ± 0.094**

Selection estimates based on viability are reproduced from Cox and Calsbeek (2010c) for comparison with selection based on RS^V^. Significant selection gradients in bold; 95% confidence intervals (95% CI) are shown below selection gradients for RS^V^.

When subadult males (SVL < 50 mm) were excluded from the dataset, patterns of selection on body size based on RS^V^ were consistent with estimates of selection that included all males (Table 2).

Selection estimates based on viability were consistent with estimates based on RS^V^ for males in 2007 and showed evidence of directional selection on male SVL (Table 2). For females, however, selection based on viability was found to be quadratic (Table 2). These results are discussed in detail in Cox and Calsbeek (2010c) and are only reproduced here for comparison.

Individual RS^V^ was highly correlated with the total number of genetic partners recorded for both males and females (χ^2^ = 547.42; *p *<* *.0001; *R* ^2^ = .87, Fig. S2). Patterns of selection on SVL based on total number of mates were consistent with estimates of selection based on RS^V^ (Table S3). Overall, selection tended to be directional when acting on male SVL (Table S3). Females experienced quadratic selection on SVL in 2006, and both directional selection and marginally significant quadratic selection in 2007 (Table S3). When data were pooled for all 3 years, males experienced positive directional selection on SVL based on number of mates (β = .21 ± .08; χ^2^ = 14.73; *p *=* *.0001) and females experienced quadratic selection on SVL based on number of mates (γ_1,1_ = −0.15 ± 0.12; χ^2^ = 4.77; *p *=* *.03).

We found no evidence of selection on hindlimb or forelimb length for either sex, when data were pooled for the 3 years of our study (Table S4). Thus, we did not pursue further tests on these traits for individual selection events by year.

## 4 Discussion

We have shown that patterns of selection based on an individual's numbers of surviving offspring are largely congruent with measures of selection based on viability in males, but patterns of selection acting on female body size are more variable among years (Table 2). Overall, we found directional selection on male body size and stabilizing selection acting on female body size. Despite the variability in the form of selection acting on female body size, these overall results are largely consistent with other studies that suggest that sexually antagonistic selection acts strongly on components of fitness related to individual reproductive success (Cox & Calsbeek, 2009; Kingsolver et al., 2001). Below, we discuss the potential mechanisms that may drive these patterns of selection.

In sexually dimorphic species, sexually antagonistic selection often acts most strongly through sexual selection (Cox & Calsbeek, 2009; Kingsolver et al., 2001). This is because reproductive roles of the sexes differ and selection acts divergently on many phenotypic traits associated with successful reproduction. In this study, we show that individual body size may be one such trait; larger males have greater numbers of mates and more offspring that survive to the next year. This could occur through processes related to either male–male competition or female choice. Brown anoles are highly territorial and males defend territories that overlap with several female territories (Stamps, 1999; Tokarz, 1995). Large male body size confers an advantage in territorial disputes (Perry & Garland, 2002; Stamps, 1988), and males that are better able to defend territories likely have greater access to mates. Thus, our results may be due to larger males having greater success at male–male competition and gaining increased reproductive success by mating with more females.

However, our measure of individual fitness also includes aspects of offspring survival, as we measured fitness in terms of the number of offspring that survived to sexual maturity. Thus, the higher fitness attributed to larger males could occur in at least two (not mutually exclusive) ways: Larger males could produce more total offspring, and/or produce offspring with higher viability. Previous studies in this system indicate that offspring sired by larger males in the laboratory have higher survival in the wild, and this result was strongest for sons (Cox & Calsbeek, 2010a). This may be related to the intralocus sexual conflict that acts on body size and females might produce more sons from larger males as a means to resolve this conflict (Calsbeek et al., 2007; Cox & Calsbeek, 2010a). Therefore, large males could have more surviving offspring both because they have greater access to mates, and because their offspring also have higher viability. Estimates of fitness such as the one used in this study have been criticized because they assign components of both parental and offspring fitness to the parent (Wolf & Wade, 2001). However, because we expect selection to act consistently on male body size based on male reproductive success and offspring survival, this fitness measure (RS^V^) best captures the total effect of selection on male body size.

We found evidence for stabilizing sexual selection acting on female body size, a pattern that is also congruent with selection measured through female viability (Cox & Calsbeek, 2010c). The same processes that confer higher survival to intermediate‐sized females may also allow them to produce more mature offspring, at least in some years. As is the case for males, access to breeding territories is also important to females, as females defend territories to gain access to preferred males and nesting sites (Stamps, 1999; Tokarz, 1998). It is possible that intermediate‐sized females are best able to hold territories, as they would be able to outcompete other females, without entering into resource competition with males. This would create a “Goldilocks” scenario (Long, Pischedda, Nichols, & Rice, 2010), in which females of intermediate size are best able to hold territories necessary for survival and reproduction.

However, female body size also exhibited positive directional selection in 1 year, possibly indicating that larger females benefit from increased access to resources and increased fecundity under some environmental conditions. For example, large body size may be favored under harsh environmental conditions when resources are limited. In these years, both males and females may experience directional selection and sexually antagonistic selection would be absent. By contrast, when environmental conditions are favorable, stabilizing selection may act on females, resulting in sexually antagonistic selection. Such a scenario would result in an environmental dependence for intralocus sexual conflict, which has been shown to occur in some insects (Berger et al., 2014) and plants (Delph et al., 2011). Future work in this system could examine whether such an environmental dependence for intralocus sexual conflict exists for brown anoles by examining whether selection on female body size varies in relation to environmental conditions. For example, this population is often adversely affected by hurricanes and droughts and such extreme weather events may play a role in selection on individual body size.

Variance in RS^V^ was statistically indistinguishable between males and females overall and for most years. This finding is somewhat surprising. Females are known to experience severe costs of reproduction (Cox & Calsbeek, 2010b; Cox et al., 2010). For example, females that are prevented from reproducing via surgical ovariectomy show higher survival (Cox & Calsbeek, 2010b), faster growth, and greater energy stores (Cox et al., 2010) compared to sham surgical controls. Given this high cost of reproduction, females should be the choosier sex (Trivers, 1972) and exhibit lower variance in reproductive success than males (Bateman, 1948). Instead, our data are consistent with the hypothesis that female promiscuity in this system is adaptive. Benefits of promiscuity may arise through the production of genetically diverse offspring (Andersson, 1994; Jennions & Petrie, 2000; Uller & Olsson, 2008), as has been recently suggested for field crickets (Rodríguez‐Muñoz, Bretman, Slate, Walling, & Tregenza, 2010) and Eastern chipmunks (Bergeron, Montiglio, Réale, Humphries, & Garant, 2012).

Although there remains much to learn regarding the specific mechanisms that drive selection on male and female body size in brown anoles, we have provided new evidence that aspects of reproductive selection on body size are sexually antagonistic. These patterns are in line with those detected through studies of viability selection. Thus, selection favors different optima in the sexes for both survival and reproductive success—two major components of fitness. This indicates that intralocus sexual conflict acting on body size is a persistent force in this system across the ontogeny of *A. sagrei* and mechanisms that resolve this conflict are likely to be favored by selection.

## Funding Information

Directorate for Biological Sciences (Grant/Award Number: DEB 0816862).

## Conflict of Interest

None declared.

## Data Accessibility

Phenotypic and genetic data used in this study will be made publicly available on DRYAD following the publication of this manuscript.
