# Supplementary material for: Field estimates of parentage reveal sexually antagonistic selection on body size in a population of Anolis lizards
Source: Ecol Evol. 2016 Sep 9;6(19):7024–31. doi: 10.1002/ece3.2443 (PMC5513217; doi:10.1002/ece3.2443)

**Supporting Information**

**Table S1**: Microsatellite primer concentrations and fluorescent tags for multiplex PCR.

| **Pool 1** | **Concentration (µM)** |
| --- | --- |
| Acar 23 F - UNI | 3 |
| Acar 23 R | 3 |
| UNI PET* | 3 |
| AAAG 61 F - HEX | 3 |
| AAAG 61 R | 3 |
| AAAG 68 F - VIC | 1 |
| AAAG 68 R | 1 |
| AAAG 70 F – 6FAM | 1 |
| AAAG 70 R | 1 |
| AAAG 91 F - PET | 2 |
| AAAG 91 R | 2 |
|  |  |
| **Pool 2** | **Concentration (µM)** |
| Acar 11 F - UNI | 2 |
| Acar 11 R | 2 |
| UNI PET* | 2 |
| AAAG 38 F - PET | 2 |
| AAAG 38 R | 2 |
| AAAG 76 F - NED | 0.75 |
| AAAG 76 R | 0.75 |
| AAAG 77 F - HEX | 0.75 |
| AAAG 77 R | 0.75 |
| AAAG 94 F – 6FAM | 1.5 |
| AAAG 94 R | 1.5 |

*UNI primers allow tagging of forward primers by annealing a fluorescent-tagged universal primer to a complimentary sequence in the forward primer.

**Table S2:** Summary statistics for microsatellite markers that were used to assign paternity, including: Number of Alleles (N_A_), Observed Heterozygosity (H_obs_), Null Allele Frequency [F(Null)], Polymorphic Information Content (PIC), combined Non-Exclusion Probability for the first parent (NE-1P), and combined Non-Exclusion Probability for the second parent (NE-2P).

| **Marker** | **N_A_** | **H_obs_** | **F(Null)** | **PIC** | **NE-1P** | **NE-2P** |
| --- | --- | --- | --- | --- | --- | --- |
| Acar 23 | 10 | 0.272 | +0.3573 | 0.526 | 0.817 | 0.661 |
| AAAG 61 | 6 | 0.444 | +0.2328 | 0.662 | 0.708 | 0.537 |
| AAAG 68 | 10 | 0.670 | +0.1070 | 0.808 | 0.508 | 0.337 |
| AAAG 70 | 8 | 0.746 | +0.0387 | 0.777 | 0.560 | 0.384 |
| AAAG 91 | 8 | 0.794 | +0.0148 | 0.795 | 0.535 | 0.360 |
| Acar 11 | 19 | 0.541 | +0.2378 | 0.865 | 0.393 | 0.244 |
| AAAG 38 | 12 | 0.744 | +0.0326 | 0.769 | 0.565 | 0.385 |
| AAAG 76 | 12 | 0.746 | +0.0865 | 0.875 | 0.377 | 0.231 |
| AAAG 77 | 7 | 0.742 | +0.0370 | 0.770 | 0.575 | 0.397 |
| AAAG 94 | 10 | 0.574 | +0.0453 | 0.605 | 0.755 | 0.575 |

**Table S3:** Linear (β) and quadratic (γ) selection gradients for selection on standardized snout-vent length (SVL) based on relative number of mates from which each individual produced offspring that survived to maturity. Significant selection gradients in bold.

| Year | Sex | *N* | Linear selection  β ± 1 SE (*P* value) | Quadratic selection  γ ± 1 SE (*P* value) |
| --- | --- | --- | --- | --- |
| 2005 | Male | 124 | 0.160 ± 0.099 (0.088) | -0.119 ± 0.146 (0.313) |
|  | Female | 112 | 0.038 ± 0.110 (0.693) | -0.047 ± 0.147 (0.784) |
| 2006 | Male | 146 | **0.204 ± 0.153 (0.019)** | 0.022 ± 0.310 (0.678) |
|  | Female | 166 | -0.018 ± 0.148 (0.814) | **-0.240 ± 0.218 (0.029)** |
| 2007 | Male | 119 | **0.288 ± 0.147 (0.005)** | 0.292 ± 0.348 (0.764) |
|  | Female | 167 | **0.216 ± 0.131 (0.008)** | -0.160 ± 0.216 (0.102) |

**Table S4:** Linear (β) selection gradients on standardized limb length based on relative RS^V^ (number of offspring that survived to maturity). Data are pooled from three years (2005-2007) to assess overall trends.

| Trait | Sex | *N* | Linear selection  β ± 1 SE (*P* value) |
| --- | --- | --- | --- |
| Hind Limb | Male | 377 | 0. 0.16 ± 0.10 (0.12) |
|  | Female | 457 | 0.035 ± 0.07 (0.59) |
| Fore Limb | Male | 377 | 0.08 ± 0.10 (0.44) |
|  | Female | 457 | 0.08 ± 0.06 (0.21) |

**
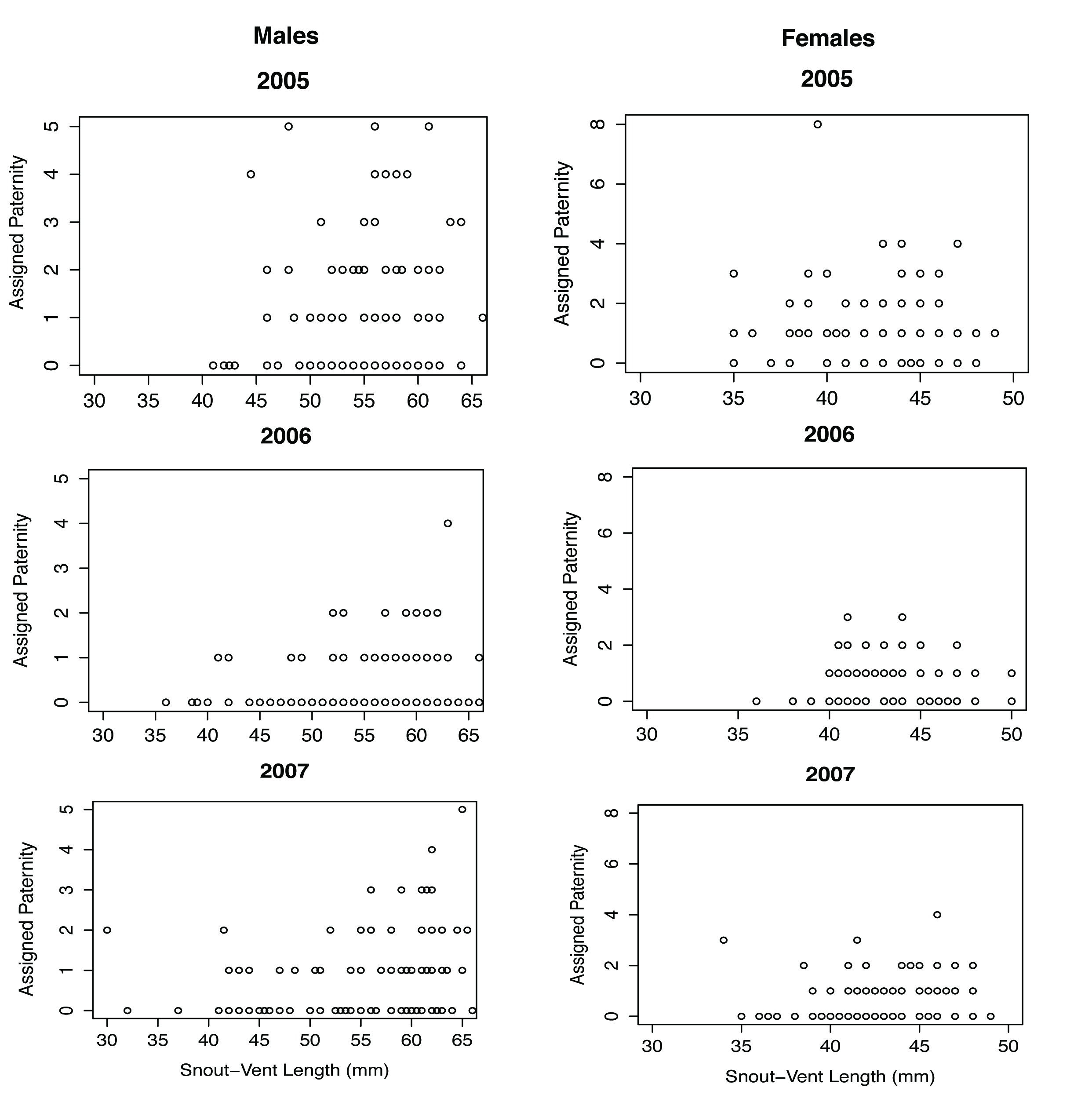
Figure S1:** Assigned parentage by individual Snout-Vent Length (mm)

**Figure S2:** Number of Mates based on genetic paternity assignment by individual Snout-Vent Length (mm)


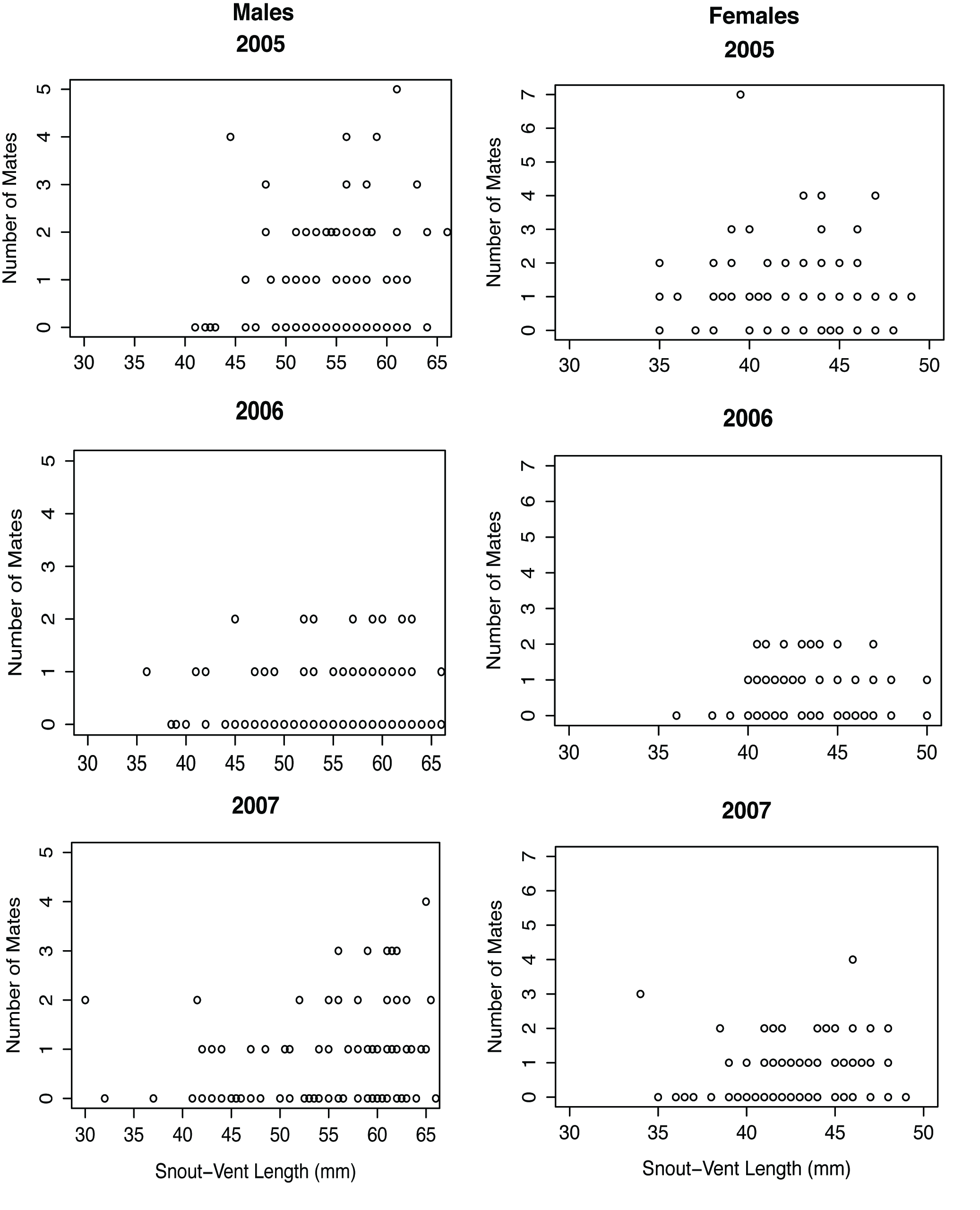

Supplement: Supplementary file 1 [file ECE3-6-7024-s001.docx]
